# Information theoretic and machine learning approaches to quantify non-linear visual feature interaction underlying visual object recognition

**DOI:** 10.1186/1471-2202-13-S1-P2

**Published:** 2012-07-16

**Authors:** Alireza Alemi-Neissi, Carlo Baldassi, Alfredo Braunstein, Andrea Pagnani, Riccardo Zecchina, Davide Zoccolan

**Affiliations:** 1 Area di Neuroscienze, Scuola Internazionale Superiore di Studi Avanzati (SISSA), Trieste, Italy; 2 Dipartimento di Scienza Applicata e Tecnologia, Politecnico di Torino, Torino, Italy; 3 Human Genetic Foundation (HuGeF), Torino, Italy

## 

Understanding what object features the visual system relies upon, when engaged in visual object recognition, is a longstanding challenge in psychophysical studies of visual perception. Although successful approaches have been developed to address this issue (e.g., *image classification* methods [1]), they have a major limitation: they can estimate the relationship between input visual images and output behavioral responses only under the assumption of a *linear observer model* (e.g., an observer performing a weighted sum of the information carried by individual pixels within an image). For instance, one popular image classification approach (named the *Bubbles* method [2]) recovers the salient features used by an observer to identify an object, by presenting the target object partially occluded by opaque masks punctured by transparent windows, and then averaging the trials yielding to correct object identification. However, this method cannot tell whether multiple salient features in an object interact non-linearly (e.g., whether those features need to be simultaneously visible for the object to be correctly identified). Here we propose two approaches to overcome this limitation.

As a preliminary step, we simulated observers with various recognition strategies. Each observer had to discriminate bubbles-masked input images of two different objects, by comparing them with either linear or non-linear object templates. One of the object templates contained two features, whose *evidence* in any given input image was computed as the dot-product between the image and the features themselves. Finally, the overall *template evidence* was computed either as: 1) the product of the two feature evidences (AND-like feature interaction); 2) the maximum of the feature evidences (OR-like feature interaction); or 3) the sum of the feature evidences (linear interaction).

The simulated observers served as benchmarks to validate two different feature-interaction analysis approaches. The first approach consisted of measuring the mutual information between the product/max/sum of every pair of pixels in the object image and the simulated observers’ responses. This approach successfully recovered the simulated feature interaction strategy, which was shown to convey significantly higher information about the observer’s response, as compared to the two other alternative strategies (permutation test; *p* < 10^6^). The second approach consisted in fitting a model to the AND-like, non-linear observer’s responses, which could predict responses to novel input images. As a model we chose a regularized logistic regression classifier, which was fed with both individual pixel values and the product of pixel pairs. The classifier successfully generalized to unseen input images (training performance=100%, test performance=87%) and its maximal weights matched/captured the simulated feature interaction (e.g., pixels’ products had maximal weights). Finally, we also started applying *sparse classifiers*[3], which, by relying on diluted discrete weights, could potentially yield a cleaner (i.e., more parsimonious) estimate of salient object features and their interaction.

In summary, our simulations show that both information theoretic and classifiers-based approaches can recover the type and the strength of the interaction among the salient features of an object. We are now working to apply these methods to analyze data obtained in a *Bubbles*-based behavioral study of rat visual object recognition.
